# CT Radiomics Models Did Not Outperform Experts in Predicting [^68^Ga]Ga-PSMA-PET Positivity in Prostate Cancer Lymph Node Staging

**DOI:** 10.3390/curroncol33030146

**Published:** 2026-03-02

**Authors:** Thula Cannon Walter-Rittel, Boris Gorodetski, Alexander Hartenstein, Julian Rogasch, Imke Schatka, Holger Amthauer, Marcus Makowski, Charlie Alexander Hamm, Tobias Penzkofer

**Affiliations:** 1 Department of Radiology, Charité Universitätsmedizin Berlin, Corporate Member of Freie Universität Berlin and Humboldt Universität zu Berlin, Augustenburger Platz 1, 13353 Berlin, Germany; boris.gorodetski@charite.de (B.G.); charlie.hamm@charite.de (C.A.H.); tobias.penzkofer@charite.de (T.P.); 2 Bayer AG, Kaiser-Wilhelm-Allee 1, 51373 Leverkusen, Germany; alexander.hartenstein@bayer.com; 3 Department of Nuclear Medicine, Charité Universitätsmedizin Berlin, Corporate Member of Freie Universität Berlin and Humboldt Universität zu Berlin, Augustenburger Platz 1, 13353 Berlin, Germany; julian.rogasch@charite.de (J.R.); imke.schatka@charite.de (I.S.); holger.amthauer@charite.de (H.A.); 4 Berlin Institute of Health, Charité Platz 1, 10117 Berlin, Germany; 5 Department of Radiology, Klinikum Rechts Der Isar, Technical University Munich, Ismaninger Str. 22, 81675 Munich, Germany; marcus.makowski@tum.de

**Keywords:** prostate cancer, lymph node metastasis, radiomics, computed tomography (CT), diagnostic imaging

## Abstract

**Background**: The use of [^68^Ga]Ga-PSMA-PET/CT for prostate cancer (PCa) staging is limited by cost and availability. This study evaluates whether radiomic features from contrast-enhanced (CE) CT can predict PSMA-positive lymph nodes (LNs) as a surrogate for metastasis. **Methods**: A retrospective study of 447 patients included 2537 segmented LNs (425 PET-positive, 2112 PET-negative). Two uroradiologists assessed 417 LNs on CE-CT using a four-point Likert scale. Radiomic features were extracted, selected using four algorithms, and analyzed with six model-building methods. Model performance was compared to radiologist ratings. **Results**: Radiomic models achieved an accuracy of 0.77–0.85, sensitivity of 0.85–0.91, and specificity of 0.74–0.85. Compared to radiologists, models had higher NPV (0.97–0.98 vs. 0.96) and sensitivity (0.85–0.91 vs. 0.76), but radiologists had superior accuracy (0.95 vs. 0.77–0.85) and specificity (0.97–0.98 vs. 0.74–0.85). In a subanalysis of LNs rated as probably benign or malignant, expert radiologists outperformed the algorithm with greater specificity and PPV (*p* < 0.005). A density threshold of >27 HU predicted PSMA-positive LNs with 0.79 accuracy, 0.87 sensitivity, and 0.78 specificity. **Conclusions**: While radiomics did not outperform expert radiologists, the single first-order parameter CT density >27 HU was predictive of PSMA-positive LNs. **Clinical Relevance Statement:** Radiomic models did not outperform expert uroradiologists. However, in high-volume or resource-limited settings lacking access to [^68^Ga]Ga-PSMA-PET/CT, they may help improve LN assessment in PCa patients with CT alone.

## Simple Summary

Contrast-enhanced CT is widely available for prostate cancer staging, whereas [^68^Ga] PSMA PET/CT—a sensitive and accurate method—is limited by cost and access. We investigated whether CT radiomics can predict PSMA-positive lymph nodes in prostate cancer patients. In 447 patients, 2537 lymph nodes were segmented (425 PSMA-positive) and several feature-selection and classification pipelines were trained and tested; two expert uroradiologists independently assessed a subset of 417 nodes on CT. Radiomics models showed good sensitivity and high negative predictive value, supporting potential use for ruling out nodal involvement, but they did not outperform expert readers overall because reader specificity and accuracy were higher. A simple CT-derived parameter was found to be predictive for PSMA-positive nodes: median lymph node attenuation, with an optimal threshold around 27 Hounsfield units, was associated with PSMA positivity and provided clinically usable discrimination. This pragmatic CT metric may aid nodal risk stratification when PSMA PET/CT or specialist interpretation is not available.

## 1. Introduction

Prostate cancer (PCa) is the most common malignancy in European men, with aggressive subtypes showing high mortality rates [1,2]. In patients with intermediate and high-risk PCa, correct nodal staging in primary staging and in the setting of biochemical recurrence is crucial, as it defines the further course of therapy [3]. ^68^Ga- or ^18^F-labelled PSMA-PET/CT has been established as the most effective modality for nodal staging in PCa with high specificity and sensitivity compared to CT/MRI [2,4,5,6].

However, PSMA-PET/CT remains inaccessible to large portions of the world, where 92–95% of low-to-lower-middle-income nations lack PET/CT units [7,8]. Even in developed countries, availability and reimbursement for PSMA-PET/CT are limited to specialized centers, and it is associated with higher costs. However, recent studies suggest that PSMA-PET/CT may still be cost-effective considering the potential downstream costs of missed metastases in CT/MRI [9].

The mere visual assessment of cross-sectional imaging processes only a small percentage of image information when reading scans [10]. Beyond the mere assessment of size and shape, CT density measurements have been used to differentiate benign from malignant LNs in PCa and may be especially helpful when evaluating PET-indeterminate LNs [5]. Also, radiomic features and models—image information extracted and modelled by computers, invisible to the human eye—have recently shown potential in the prediction of lymph node (LN) metastasis in different types of cancer, including PCa [11,12]. Likewise, models combining radiomic features of PSMA-PET, CT and clinical data have also shown promising results in predicting metastatic disease in PCa [13].

These recent findings inform the purpose of this study to evaluate whether radiomic features and models based on CT images can sufficiently predict LN metastasis in PCa. Radiomic features might be able to add additional imaging biomarkers to conventional CT in PCa to overcome the shortcomings of the method in nodal staging at a lower cost compared to PSMA-PET/CT with a potential offset difference in diagnostic performance.

It was our hypothesis that the prediction of PSMA-positive metastatic LN in PCa can be improved with textural features extracted from contrast-enhanced (CE) CT.

## 2. Materials and Methods

### 2.1. Study Design and Population

This single-center, retrospective study was approved by the institutional ethics review board (Ethikkommission Charité Berlin) and was conducted ethically in accordance with the World Medical Association Declaration of Helsinki. Written informed consent was waived due to the study’s retrospective design.

Inclusion required a [^68^Ga]Ga-PSMA PET/CT with diagnostic venous-phase CE-CT. ‘Baseline’ was defined as the first PET/CT per patient within the study period (primary staging or restaging) and patient age >18 years; subsequent follow-up PET/CT examinations were excluded. Exclusion criteria were (1) lack of CE-CT, (2) insufficient/non-diagnostic image quality of PET and or CE-CT, (3) follow-up [^68^Ga]Ga-PSMA-PET/CT scan (only baseline PSMA-PET/CT—i.e., first in a series—included).

### 2.2. [^68^Ga]Ga-PSMA PET/CT Imaging Protocol

[^68^Ga]Ga-PSMA PET/CT imaging was performed on a Gemini Astonish TF 16 Scanner (Philips Medical Systems, Best, The Netherlands). A low-dose CT was performed for anatomical mapping and attenuation correction prior to the PET scan. PET data was acquired approx. 65 min (range 58–70 min) after intravenous injection of 150–250 MBq [^68^Ga]Ga-PSMA-HBED-CC. A diagnostic CE-CT was acquired in the venous phase immediately after completion of the PET-scan.

### 2.3. Patient Cohort and Segmentation

447 patients (68.7 ± 7.54 (45–87) years, PSA 20.9 ± 94.6 (0–1423) ng/mL) who had received [^68^Ga]Ga-PSMA PET/CT with CE-CT exam between September 2009 and April 2017 met the inclusion criteria. Of 447 included patients, 111 underwent pretreatment imaging and 336 underwent PET/CT for re-staging. The CE-CT was performed in the venous phase after IV contrast immediately after acquisition of the PET scan. A total of 2537 LNs were identified and segmented (425 were PET-positive, 2112 were PET-negative), with 0.83 positive and 3.9 negative nodes per patient on average.

LN segmentation was performed semi-automatically with MITK 2016 3.0 (German Cancer Research Center, Division of Medical Image Computing, Heidelberg, Germany) with manual correction on venous-phase CE-CT in the axial plane. For each patient, a maximum of five LNs were selected and segmented. Although overall, larger LNs were preferred over smaller ones and obvious metastases were preferred over overtly benign LNs, the median short axis diameter (SAD) of the segmented LNs in this study was 7.1 mm (SD +/− 4.3 mm).

### 2.4. Data Curation and Annotation

A radiologist and a nuclear medicine specialist (more than five years of experience with PSMA PET/CT) classified the respective LNs as malignant or benign. [^68^Ga]Ga-PET-CT image data was used as the ground truth. Any focal increase in uptake of [^68^Ga]Ga-PSMA with an SUVmax of >2.5 that could not be associated with physiological uptake or background was considered as malignant tumor infiltration of the LNs. In cases where the SUVmax was >2.0 and <2.5, the uptake was evaluated by a second nuclear medicine specialist to exclude physiological uptake or background. Overall, we used an approach as described in several previous studies [14,15].

### 2.5. Data Extraction and Partitioning

The data set (2537 LNs) was divided into a training set of 2120 (357, 17%, PET-positive and 1763, 83%, PET-negative LNs) and an internal validation test set of 417 (68, 16%, PET-positive and 349, 84%, PET-negative LNs). A further training set was created and balanced with the 357 PET-positive LNs and 357 randomly matched PET-negative LNs to prevent both overfitting and underfitting.

First radiomics features were extracted using the pyradiomics package (Version 2.0.0, https://github.com/Radiomics/pyradiomics, accessed 5 April 2021) [16]. Imaging data was resampled to isotropic voxels 1.0 × 1.0 × 1.0 mm using the sitkBSpline interpolator [17]. The following groups of radiomics features were used: shape, first order, glcm, gldm, glrlm and glszm. Binning of voxel values with a bin width of 25 HU was used for feature stabilization [16]. After feature extraction, feature selection was performed.

Four feature selection methods were used: Wilcoxon, Receiver-Operator-Curves (ROC), mutual information (MI), and maximum relevance minimum redundancy (MRMI). Features were reduced to *n* = 20 to avoid overfitting. After feature selection, six models were created and compared: linear discriminant analysis (LDA), logistic regression (LR), partial least squares (PLS), support vector machines (SVM), multilayer perceptron (MLP), and recursive partition (RPART). RPART classification trees were extracted for each feature selection method (Wilcoxon, ROC, mutual information, MRMI).

All models were tested with the internal validation test set (417 LNs) and compared to two expert radiologists with at least 5 years of experience in urogenital imaging (R1 + R2). The readers (R1 + R2) were presented with an 80 × 80 × 80 mm^3^ volume centered on the target LN with a resolution of 1 × 1 × 1 mm^3^ and asked to categorize the likelihood of LN infiltration on a Likert scale:

- 0—very likely benign LN
- 1—probably benign LN
- 2—probably malignant LN
- 3—very likely malignant LN

The model performance was compared to these ratings.

In a second step, the model performance was compared to the radiologists in a setting where expert radiologists were unsure of the diagnosis (i.e., LNs rated as “likely benign” or “likely malignant”). In this compound model the ratings were aggregated as follows:

- 1—very likely benign and probably benign LN
- 2—very likely malignant and probably malignant LN

**Figure 1:**
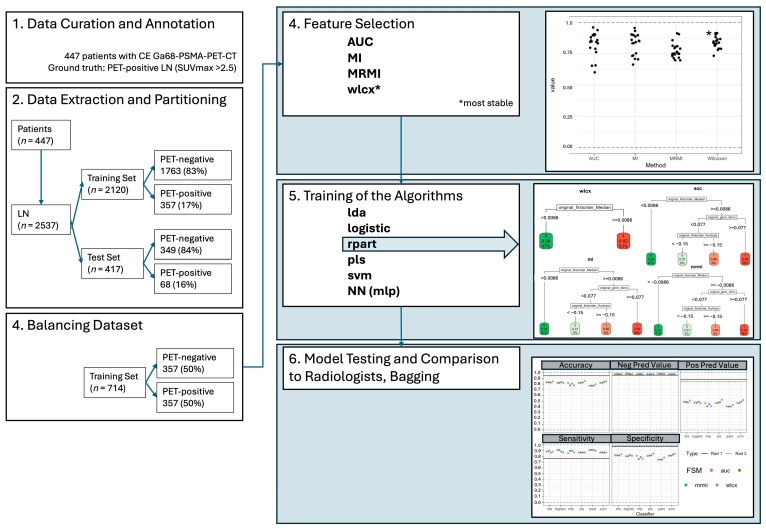
Illustration of methodology—Part 1—Overview of methods part one with data extraction, partitioning, curation of the test and validation data sets as well as the various feature selection methods, the illustration of investigated different algorithms and the resulting decision trees with RPART and the “first_order_median” (median HU) feature as well as the model testing and comparison with radiologists.

To counter potential bias through random balancing, the training and test sets were bootstrapped in twenty iterations and the results were aggregated; see Figure 1.

### 2.6. Statistical Analysis

To enable binary output for model comparisons, the optimal threshold for positive output was determined by maximizing Youden’s index (Sensitivity + Specificity − 1). Accuracy, sensitivity, specificity, PPV and NPV were calculated using binary predictions. All statistical analyses were performed by an expert statistician in R 3.6.1 (R Core Team, 2019) [18] using the add-on packages knitr, readxl, tidyverse, mRMRe, caret, MASS, rpart, rpart.plot, pROC, kableExtra, pls, glmnet, rms, pROC, pathwork, and DescTools. Comparison between radiologists and model performance were carried out using logistic regression; in case of non-overlapping 95% confidence intervals, a statistically significant difference at α = 0.05 was assumed.

## 3. Results

### 3.1. Feature Selection Results

The feature selection process successfully reduced the number of radiomic features while retaining key metrics. Seven features were selected consistently across all four methods: firstorder_Median, firstorder_Mean, firstorder_90Percentile, firstorder_10Percentile, gldm_LowGrayLevelEmphasis, gldm_DependenceNonUniformityNormalized, and glcm_Idmn. These features represent a combination of first-order intensity and texture features. Nine features were selected by three methods, including additional first-order features such as firstorder_TotalEnergy, firstorder_Skewness, firstorder_Maximum, and firstorder_Kurtosis, alongside texture metrics such as glrlm_ShortRunLowGrayLevelEmphasis, glrlm_LongRunHighGrayLevelEmphasis, gldm_SmallDependenceLowGrayLevelEmphasis, gldm_LargeDependenceHighGrayLevelEmphasis, and glcm_Idn. Short axis diameter of the respective LN was not included in the extracted features.

The Wilcoxon (WLCX) method demonstrated the highest stability among the feature selection techniques, as observed in simulations involving random dataset splits (mean overlap = 0.842, SD = 0.041). Stability values for other methods were slightly lower: AUC with a mean overlap of 0.835 (SD = 0.088), MI with 0.835 (SD = 0.087), and MRMI with 0.772 (SD = 0.060). See also Figure 1 and Supplemental Table S1.

### 3.2. Single Parameter Performance: Median Hu Value

**Figure 2:**
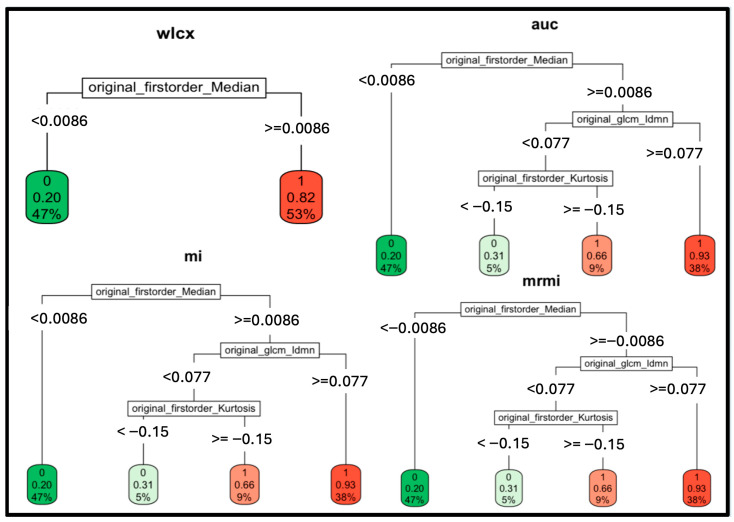
Illustration of RPART classification trees with the variable feature selection methods with the “original_firstorder_Median” = median HU of the lymph nodes. The first order median HU value reveals a good performance for a cut-off at a median HU value when recalculated from the source data with the following formula (assuming a standard deviation of 31.4): −0.0086 × 31.4 + 27.02 = 26.75; i.e., a median HU value of 27. Wlcx—Wilcoxon; auc—area under the curve; mi—minimum information: mrmi—maximum relevance minimum redundancy.

The firstorder_Median feature was identified as the dominant predictor by the RPART method in all four feature selection methods, with a parameter cut-off even for a single feature of −0.0086, which can safely be rounded to 0. A cut-off value of 27 (median_hu < 27: benign, median_hu ≥ 27: malignant) was achieved after inverse normalization. This threshold was validated using an independent test set of 416 lymph nodes, where classification based on the median HU value alone achieved an accuracy of 0.79, sensitivity of 0.87, and specificity of 0.78. Positive predictive value (PPV) and negative predictive value (NPV) were 0.43 and 0.97, respectively. See also Figure 1 and Figure 2.

### 3.3. Calibration and Brier Scores

**Figure 3:**
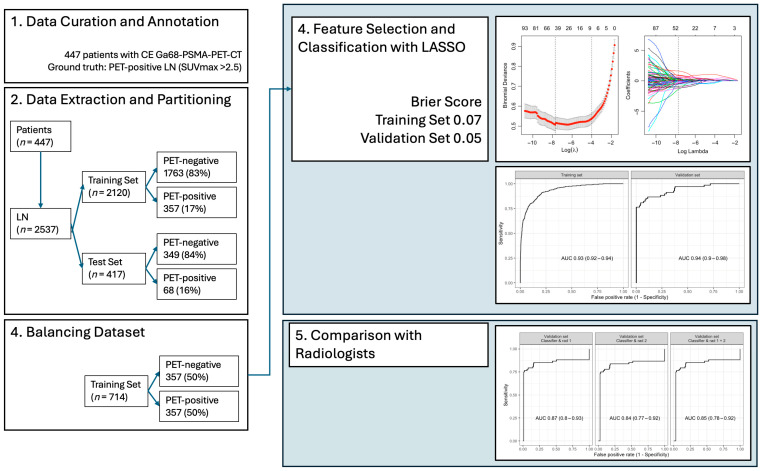
Illustration of methodology—Part 2—Overview of methods part two with data extraction with the LASSO method. Comparison of the extracted classifiers with two expert radiologists.

Calibration plots for the training set showed acceptable alignment between predicted and observed probabilities, with minor deviations in probabilities ranging between 0.25 and 0.75. In the independent test set, predicted probabilities deviated more substantially in the mid-range values. The Brier score for the training set was 0.07, while the independent test set achieved a Brier score of 0.05, indicating good model calibration for binary classification; see also Figure 3.

### 3.4. Confusion Matrix Analysis

Using the independent test set, confusion matrices revealed a sensitivity of 0.868 and specificity of 0.775 when using the optimal cut-off derived from training data (0.13). When the threshold was adjusted to 0.42 based on test set predictions, accuracy increased to 0.933, sensitivity decreased to 0.809, and specificity improved to 0.957. These metrics suggest that optimal thresholds vary based on dataset characteristics.

### 3.5. Model Robustness and Performance

#### 3.5.1. Model Performance on the Independent Test Set

The applied models using different classifiers and feature selection methods showed consistent accuracy (0.77–0.85), sensitivity (0.85–0.91), and specificity (0.74–0.85) on the independent test set. The highest balanced accuracy was observed with models trained using the WLCX method (Figure 3). When comparing classifiers, the following ranges of performance metrics were recorded across the independent test set (Figure 1; Supplemental Table S1):

- LDA: ACC = 0.83–0.85, Sensitivity = 0.87–0.90, Specificity = 0.82–0.85.
- LR: ACC = 0.82–0.83, Sensitivity = 0.87–0.91, Specificity = 0.80–0.82.
- SVM: ACC = 0.82–0.84, Sensitivity = 0.87, Specificity = 0.81–0.83.
- MLP: ACC = 0.77–0.80, Sensitivity = 0.85–0.91, Specificity = 0.74–0.79.
- RPART: ACC = 0.77–0.80, Sensitivity = 0.87–0.91, Specificity = 0.74.

#### 3.5.2. Comparison with Expert Radiologists

Interobserver agreement between the two readers was moderate (Cohen’s Kappa: 0.505).

In a binary aggregation of LN classifications (0/1—benign vs. 2/3—malignant), the different evaluated models showed similar NPV (models: 0.97—0.98; radiologists: 0.96), superior sensitivity (models: 0.85—0.91 vs. radiologists: 0.76) and worse ACC (models: 0.77–0.85 vs. radiologists 0.95) and specificity (models: 0.74–0.85; radiologists: 0.97–0.98; *p* < 0.05) compared to expert radiologists. The PPV for each of the models (0.41–0.53) was significantly lower compared to the expert readers (0.84–0.88, *p* < 0.05); see Figure 1, Table 1 and Table 2.

**Figure 4:**
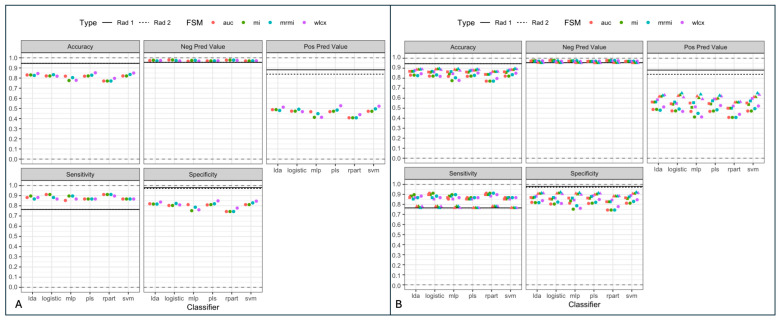
Model comparison with expert radiologists—Comparison of the extracted classifiers with two expert radiologists (dashed and black horizontal line) compared to the various classifiers identified with the four different feature selection methods (FSM). Left (**A**): a dichotomized setting of “definitely benign” vs. “definitely malignant” nodes. Right (**B**): a setting of uncertainty with “likely benign” vs. “likely malignant” LNs. Sensitivity and negative predictive value (NPV) are improved in the first setting (**A**), otherwise the radiologists outperform the classifiers. In a setting of uncertainty (**B**) the performance of the classifiers is reduced (squares and triangles represent the performance metrics in the setting of uncertainty).

When stratified by intermediate classifications (1 = probably benign, 2 = probably malignant), models exhibited a decline in sensitivity and specificity compared to binary classifications; see Figure 4. For cases classified as intermediate, model performance metrics decreased by an average of 10% across sensitivity, specificity, and accuracy (Supplemental Table S2).

## 4. Discussion

This study confirms that radiomics parameters from CE-CT may aid in identifying metastatic LNs in PCa using [^68^Ga]Ga-PSMA PET/CT as a pragmatic reference. Our principal finding is a clinically relevant performance trade-off: across multiple modelling strategies, radiomics achieved consistently high sensitivity and NPV, while expert uroradiologists maintained substantially higher specificity, PPV, and overall accuracy.

From a clinical perspective, sensitivity and NPV are important since missed nodal metastases may alter treatment intent. In that sense, a sensitivity-focused model may offer value as a triage or “second-reader” tool, especially in high-volume environments or where access to expert uroradiologists may be limited and provided it is not used as a stand-alone method for upstaging.

Conversely, experts’ superior specificity/PPV reflects the continued importance of experienced pattern recognition to avoid false positives that could trigger unnecessary downstream imaging or treatment escalation. We therefore interpret our results as supportive of radiomics as a decision support tool, not as a replacement for expert assessment.

A second important finding of our study is that radiomics parameters, especially those analyzing non-morphological aspects like CT density in Hounsfield Units (HU) of lymph nodes (LNs) on CE-CT, can improve the discriminative power of CT to identify malignant LNs in prostate PCa. A cut-off value of 27 HU was helpful in identifying malignant (PSMA-positive) LNs. This cut-off value is quick and easy to implement in clinical routine, for example, when reading a CT with equivocal LNs in patients with PCa.

Interestingly, the “classic” morphologic parameters (short axis diameter (SAD), maximum diameter in 2D) did not perform as well as CT HU-based parameters. Our findings are in keeping with previous studies which found that malignant LNs had a higher density compared to non-malignant LNs in lung cancer patients as well as other primary cancers, including PCa; both studies found a HU threshold between 7.5 HU and 20 HU on non-enhanced CT [5,19]. A post-contrast density cut-off might be helpful in a clinical setting where routine staging is typically performed in the venous phase. While promising, this cut-off should not be used in isolation for clinical decision-making but rather considered alongside other clinical and imaging factors. Also, given that attenuation may vary depending on acquisition and contrast timing, this further validation in larger, multi-center studies is required before widespread clinical implementation.

Other previous studies have also evaluated the usefulness of morphologic features for LN classification, yielding AUC values of up to 0.76 for single parameters such as LN volume or short axis diameter or sphericity for tumor entities other than PCa [20,21]. However, standard morphological features alone fall short in PCa, where approximately 80% of metastatic LNs are smaller than 8 mm in short axis diameter [22,23].

Overall, the radiomics models, which included further first and second order features derived from CE-CT that were not investigated here, did not outperform two expert radiologists, even after exhaustive and comprehensive modelling and feature extraction. Our findings contrast with previous studies on CT-based radiomics features from [^68^Ga]PSMA-PET CT, as described by Peeken et al., who found that their model outperformed conventional morphologic metrics such as LN SAD and LN volume, as well as expert readers [12]. It is of note that Peeken et al. included only patients with histologically proven LN metastasis with much greater mean SAD, as opposed to the study presented here which not only included more LNs, but in which a majority of LNs considered were non-metastatic and much smaller in size. However, including non-metastatic LNs in the evaluation of predictive radiomics models is important to prevent positive bias.

Even though the models were not superior to expert radiologists in our study, given the speed and consistency at which a model would predict malignant LNs in PCa, they may yet be helpful to standardize diagnostic performance. This would be particularly helpful in high-volume settings and for less experienced radiologists.

In this study, interobserver agreement between the two expert readers was moderate (Cohen’s κ = 0.505) consistent with the known challenge of CT-based nodal staging in predominantly sub-centimeter metastases [24,25]. Considering this interobserver variability, the model might also help to improve standardization in the setting of PCa, where most LNs without increased SAD are still likely to be benign.

Overall, the model is best positioned as a decision support tool. A feasible workflow is to compute median HU (and/or a radiomics risk score) for radiologist-identified nodes on venous-phase CE-CT and flag nodes above the validated threshold for prioritized review, structured reporting, and consideration of [^68^Ga]PSMA PET/CT or targeted management. Given the model’s high sensitivity/NPV but lower specificity compared with experts, implementation should focus on triage/standardization rather than automated upstaging, and should include local calibration and prospective performance monitoring.

There are a few limitations to our study. Firstly, this study is a single-center, single-scanner study, albeit with a large patient and LN cohort. On the one hand, this allowed us to develop a stable, reproducible method, while on the other hand this also limits the generalizability of the method. While the imaging protocol was consistent in our study, multicenter deployment will encounter variability in scanners, reconstruction kernels, contrast timing, and dose, all of which may alter radiomic feature distributions and calibration. In this setting, harmonization strategies become important: protocol alignment, locked preprocessing (e.g., isotropic resampling), and statistical harmonization or recalibration when pooling heterogeneous data.

What is more, [^68^Ga]Ga-PSMA-PET/CT was used as the gold standard for detecting LN metastases, rather than histopathology, which is the definitive method for diagnosis. While [^68^Ga]PSMA-PET/CT is highly sensitive and specific for detecting LN metastases in PCa, histopathological examination remains the definitive diagnostic method [2]. Although [^68^Ga]PSMA PET/CT shows high diagnostic accuracy for nodal disease, PSMA uptake interpretation is subject to physiological uptake patterns and pitfalls, and there is no universally accepted absolute SUVmax threshold for nodal malignancy. We used an operational rule (SUVmax > 2.5 with adjudication for equivocal uptake) to enable consistent labelling in a large retrospective cohort. However, verification bias remains possible, particularly for small nodes where partial-volume effects may reduce measured uptake. Future studies incorporating histopathological validation could provide more robust assessments of radiomic model performance, but would also risk selection bias as benign LNs would be underrepresented.

Also, segmentation and node sampling may introduce spectrum bias. Since a maximum of five LNs per patient were segmented and obviously malignant LNs were prioritized, the sampled distribution may underrepresent the most subtle borderline nodes. However, the LNs included in this study had a median SAD of 7.1 mm, which is well below the typical radiological cut-off used for metastatic LNs in CT. This is clinically relevant because indeterminate LNs are the real-world problem; consistent with this, model performance decreased in the “uncertainty setting” when comparing likely benign vs. likely malignant nodes. Future studies should adopt systematic sampling strategies or automated detection/segmentation to better capture the full spectrum of nodal appearances and to quantify segmentation reproducibility (e.g., feature ICC across repeated contours).

Also, our study used only experienced expert radiologists for model comparison and testing. Another avenue that might warrant exploration could be testing and model validation with less experienced radiologists to evaluate the efficacy of this method in a more real-world setting, particularly in regions where there is a shortage of radiologists [26].

Finally, there are inherent shortcomings of the radiomics method, like limited feature stability, potential overfitting and challenges in standardization [10]. Hence, implementation requires external multicenter validation before clinical use, including transportability testing across vendors and protocols, threshold recalibration, and prospective evaluation of clinical impact (e.g., changes in downstream imaging, management decisions, and false-positive burden).

One obvious solution to these issues is the application of deep learning (DL) segmentation, given the method’s rapid implementation in both segmentation, evaluation and analysis in medical imaging [27,28,29,30]. Indeed, the application of DL on the same dataset outperformed two expert radiologists in the prediction of metastatic LNs in patients with PCa [31]. Still, the radiomic method holds value, as it is easier to interpret for humans than output from DL models. Therefore, a potential trade-off occurs when using DL models, since a gain in predictive performance would come with a loss in interpretability, as DL methods output numerical predictions of tumor infiltration without any clues as to how or why a prediction was made.

Given the inherent pros and cons of both radiomic (i.e., generic) models and DL models and the fact that DL models require less human input, a successful application of both methods in a fused model might provide more robust and reproducible results [32].

## 5. Conclusions

Even after exhaustive analysis of different models based on radiomics features, radiomics did not outperform expert radiologists in predicting PSMA-positive LNs in PCa staging. However, where access to [^68^Ga]Ga-PSMA-PET/CT and expert radiologists is limited, this method and CT-density measurements of LNs may still be able to assist the comprehensive assessment of PCa patients with CT alone.

## Figures and Tables

**Table 1:** Diagnostic performance of the classifiers and respective feature selection methods. Summary of diagnostic performance of the classifiers and the respective feature selection method (FSM).

Classifier	FSM	Accuracy	Sensitivity	Specificity	PPV	NPV
LDA	WLCX	0.84	0.88	0.84	0.51	0.97
LDA	AUC	0.83	0.88	0.82	0.49	0.97
LDA	MI	0.83	0.9	0.82	0.49	0.98
LDA	MRMI	0.83	0.87	0.82	0.48	0.97
LR	WLCX	0.82	0.87	0.81	0.47	0.97
LR	AUC	0.82	0.91	0.8	0.47	0.98
LR	MI	0.82	0.91	0.8	0.47	0.98
LR	MRMI	0.83	0.88	0.82	0.49	0.97
PLS	WLCX	0.85	0.87	0.85	0.53	0.97
PLS	AUC	0.82	0.87	0.81	0.47	0.97
PLS	MI	0.82	0.87	0.81	0.47	0.97
PLS	MRMI	0.83	0.87	0.82	0.48	0.97
SVM	WLCX	0.85	0.87	0.85	0.52	0.97
SVM	AUC	0.82	0.87	0.81	0.47	0.97
SVM	MI	0.82	0.87	0.81	0.47	0.97
SVM	MRMI	0.84	0.87	0.83	0.5	0.97
MLP	WLCX	0.78	0.87	0.76	0.41	0.97
MLP	AUC	0.82	0.85	0.81	0.47	0.97
MLP	MI	0.78	0.9	0.75	0.41	0.97
MLP	MRMI	0.8	0.9	0.79	0.45	0.98
RPART	WLCX	0.8	0.9	0.78	0.44	0.98
RPART	AUC	0.77	0.91	0.74	0.41	0.98
RPART	MI	0.77	0.91	0.74	0.41	0.98
RPART	MRMI	0.77	0.91	0.74	0.41	0.98

LDA—linear discriminant analysis; LR—logistic regression; PLS—partial least squares; SVM—support vector machines (SVM); MLP—multilayer perceptron; RPART—recursive partition. WLCX—Wilcoxon; AUC—area under the curve; MI—mutual information; MRMI—maximum relevance minimum redundancy. PPV—positive predictive value; NPV—negative predictive value.

**Table 2:** Diagnostic performance of two expert radiologists.

	Accuracy	Sensitivity	Specificity	PPV	NPV
Reader 1	0.95	0.76	0.98	0.88	0.96
Reader 2	0.95	0.76	0.97	0.84	0.96

PPV—positive predictive value; NPV—negative predictive value.

## Data Availability

The datasets generated and/or analyzed during the current study are not publicly available due to data protection restrictions (GDPR) but are available from the corresponding author on reasonable request.

## Supplementary Materials

The following supporting information can be downloaded at: https://www.mdpi.com/article/10.3390/curroncol33030146/s1, Table S1: Overview of selected radiomics features with the different feature-selection methods. The Wilcoxon method (WLCX) was the most stable, indicated by an *. ROC-AUC—receiver-operator curve- area under the curve; MI—minimum information: MRMI—maximum relevance minimum redundancy; Table S2: Diagnostic performance of the classifiers and respective feature selection methods applied to LNs which were classified as likely benign or likely malignant. Summary of diagnostic performance of the classifiers and the respective feature selection method (FSM) when applied to LNs that were likely benign or likely malignant.

## Abbreviations

The following abbreviations are used in this manuscript:

ACC	accuracy
CT	computed tomography
HU	Hounsfield unit
LDA	linear discriminant analysis
LN	lymph node
LR	logistic regression
MI	mutual information
MLP	multilayer perceptron
MRMI	maximum relevance minimum redundancy
NPV	negative predictive value
PCa	prostate cancer
PET	positron emission tomography
PLS	partial least squares
RPART	recursive partition
PPV	positive predictive value
PSMA	prostate-specific membrane antigen
ROC	receiver-operating characteristic curves
SVM	support vector machines
WLCX	Wilcoxon
