# Supplementary material for: CT Radiomics Models Did Not Outperform Experts in Predicting [68Ga]Ga-PSMA-PET Positivity in Prostate Cancer Lymph Node Staging
Source: Curr Oncol. 2026 Mar 2;33(3):146. doi: 10.3390/curroncol33030146 (PMC13025960; doi:10.3390/curroncol33030146)
Supplement: Supplementary file 1 [file curroncol-33-00146-s001.zip › curroncol-4163019-supplementary.pdf]

Supplemental Material

Supplemental Table S1 – Overview of selected radiomics features with the different feature-selection methods. The Wilcoxon method (WLCX) was the most stable, indicated by an *. ROC-AUC - receiver-operator curve- area under the curve; MI – minimum information: MRMI – maximum relevance minimum redundancy.

WLCX*	ROC-AUC	MI	MRMI
firstorder_Median	firstorder_Median	firstorder_Median	firstorder_Median
firstorder_Mean	firstorder_Mean	firstorder_Mean	firstorder_Kurtosis
glcm_Idn	firstorder_10Percentile	firstorder_10Percentile	glrlm_LongRunHighGrayLevelEmphasis
glcm_Idmn	firstorder_Skewness	firstorder_Kurtosis	firstorder_10Percentile
firstorder_10Percentile	firstorder_Maximum	firstorder_Skewness	firstorder_Skewness
firstorder_90Percentile	glcm_Idn	firstorder_Maximum	glcm_Idmn
gldm_DependenceNonUniformityNormalized	glcm_Idmn	glcm_Idn	glszm_LargeAreaHighGrayLevelEmphasis
shape_SurfaceVolumeRatio	firstorder_90Percentile	glcm_Idmn	firstorder_Maximum
glrlm_RunLengthNonUniformityNormalized	firstorder_Kurtosis	firstorder_90Percentile	firstorder_TotalEnergy
glrlm_RunPercentage	glrlm_LongRunHighGrayLevelEmphasis	glrlm_LongRunHighGrayLevelEmphasis	firstorder_Mean
glcm_Idm	firstorder_Energy	firstorder_Energy	firstorder_RootMeanSquared
glrlm_ShortRunEmphasis	firstorder_TotalEnergy	firstorder_TotalEnergy	gldm_LowGrayLevelEmphasis
glszm_ZoneEntropy	gldm_LargeDependenceHighGrayLevelEmphasis	gldm_LargeDependenceHighGrayLevelEmphasis	gldm_DependenceNonUniformityNormalized
glcm_Id	glrlm_ShortRunLowGrayLevelEmphasis	glszm_LargeAreaHighGrayLevelEmphasis	glszm_ZoneVariance
glrlm_ShortRunLowGrayLevelEmphasis	gldm_DependenceNonUniformityNormalized	gldm_DependenceNonUniformityNormalized	firstorder_90Percentile
firstorder_InterquartileRange	gldm_LowGrayLevelEmphasis	glrlm_ShortRunLowGrayLevelEmphasis	glszm_SmallAreaHighGrayLevelEmphasis
gldm_DependenceVariance	gldm_SmallDependenceLowGrayLevelEmphasis	gldm_LowGrayLevelEmphasis	gldm_SmallDependenceLowGrayLevelEmphasis
gldm_LowGrayLevelEmphasis	gldm_GrayLevelNonUniformity	gldm_GrayLevelNonUniformity	glcm_MaximumProbability
glrlm_LowGrayLevelRunEmphasis	firstorder_RootMeanSquared	gldm_SmallDependenceLowGrayLevelEmphasis	gldm_LargeDependenceHighGrayLevelEmphasis
shape_MinorAxis	glrlm_LowGrayLevelRunEmphasis	glszm_ZoneVariance	firstorder_InterquartileRange

Supplemental Table s2 – Diagnostic performance of the Classifiers and respective Feature Selection Methods applied to LNs which were classified as likely benign or likely malignant. Summary of diagnostic performance of the classifiers and the respective feature selection method (FSM), when applied to LN that were likely benign or likely malignant.

Classifier	FSM	Accuracy	Sensitivity	Specificity	PPV	NPV
LDA	WLCX	0.82	0.88	0.81	0.47	0.97
LDA	AUC	0.82	0.90	0.80	0.47	0.98
LDA	MI	0.81	0.88	0.79	0.45	0.97
LDA	MRMI	0.83	0.87	0.82	0.48	0.97
LR	WLCX	0.81	0.88	0.79	0.45	0.97

LR	AUC	0.81	0.90	0.79	0.46	0.98
LR	MI	0.82	0.91	0.79	0.47	0.98
LR	MRMI	0.81	0.87	0.80	0.46	0.97
PLS	WLCX	0.82	0.88	0.81	0.47	0.97
PLS	AUC	0.81	0.88	0.79	0.45	0.97
PLS	MI	0.80	0.88	0.78	0.44	0.97
PLS	MRMI	0.82	0.87	0.81	0.48	0.97
SVM	WLCX	0.82	0.87	0.81	0.47	0.97
SVM	AUC	0.81	0.88	0.79	0.45	0.97
SVM	MI	0.81	0.88	0.79	0.45	0.97
SVM	MRMI	0.81	0.87	0.80	0.46	0.97
MLP	WLCX	0.82	0.87	0.81	0.47	0.97
MLP	AUC	0.79	0.90	0.77	0.43	0.97
MLP	MI	0.77	0.87	0.75	0.41	0.97
MLP	MRMI	0.83	0.85	0.82	0.48	0.97
RPART	WLCX	0.76	0.87	0.74	0.39	0.97
RPART	AUC	0.82	0.87	0.81	0.48	0.97
RPART	MI	0.82	0.87	0.81	0.48	0.97
RPART	MRMI	0.83	0.88	0.82	0.48	0.97

LDA - Linear discriminant analysis; linear discriminant analysis; LR - logistic regression; PLS - partial least squares; SVM - support vector machines (SVM); MLP - multilayer perceptron; RPART - recursive partition. WLCX – Wilcoxon; AUC – Area under the Curve; MI – Mutual Information; MRMI – maximum relevance minimum redundancy. PPV – positive predictive value; NPV – negative predictive value.
